# Dynamic contrast-enhanced QSM for perfusion imaging: a systematic comparison of Δ*R*2*- and QSM-based contrast agent concentration time curves in blood and tissue

**DOI:** 10.1007/s10334-020-00831-x

**Published:** 2020-02-20

**Authors:** Emelie Lind, Linda Knutsson, Freddy Ståhlberg, Ronnie Wirestam

**Affiliations:** 1 Department of Medical Radiation Physics, Lund University, Barngatan 4, 22185 Lund, Sweden; 2 Russell H. Morgan Department of Radiology and Radiological Science, Johns Hopkins University School of Medicine, Baltimore, MD USA; 3 Department of Clinical Sciences Lund, Diagnostic Radiology, Lund, Sweden; 4 Lund University Bioimaging Center, Lund University, Lund, Sweden

**Keywords:** Cerebrovascular circulation, Contrast agents, Magnetic resonance imaging, Magnetometry, QSM

## Abstract

**Objective:**

In dynamic susceptibility contrast MRI (DSC-MRI), an arterial input function (AIF) is required to quantify perfusion. However, estimation of the concentration of contrast agent (CA) from magnitude MRI signal data is challenging. A reasonable alternative would be to quantify CA concentration using quantitative susceptibility mapping (QSM), as the CA alters the magnetic susceptibility in proportion to its concentration.

**Material and methods:**

AIFs with reasonable appearance, selected on the basis of conventional criteria related to timing, shape, and peak concentration, were registered from both Δ*R*2* and QSM images and mutually compared by visual inspection. Both Δ*R*2*- and QSM-based AIFs were used for perfusion calculations based on tissue concentration data from Δ*R*2*as well as QSM images.

**Results:**

AIFs based on Δ*R*2* and QSM data showed very similar shapes and the estimated cerebral blood flow values and mean transit times were similar. Analysis of corresponding Δ*R*2* versus QSM-based concentration estimates yielded a transverse relaxivity estimate of 89 s^−1^ mM^−1^, for voxels identified as useful AIF candidate in Δ*R*2* images according to the conventional criteria.

**Discussion:**

Interestingly, arterial concentration time curves based on Δ*R*2* versus QSM data, for a standard DSC-MRI experiment, were generally very similar in shape, and the relaxivity obtained in voxels representing blood was similar to tissue relaxivity obtained in previous studies.

## Introduction

Dynamic susceptibility contrast magnetic resonance imaging (DSC-MRI) is a common MRI technique for assessment of brain perfusion and perfusion-related parameters. For estimations of cerebral blood flow (CBF), cerebral blood volume (CBV), and mean transit time (MTT) using DSC-MRI, the concentration of an exogenous contrast agent (CA) is normally determined using the effect of the CA on the transverse relaxation time. In gradient-echo (GRE) DSC-MRI, the increase in *R*2* caused by the CA is conventionally assumed to be proportional to the concentration of CA. Also, the transverse relaxivity *r*2*, describing the linear relationship between the concentration and the change in relaxation rate, Δ*R*2* has, as a first approximation, been assumed to be the same for blood and all types of brain tissue [1]. However, it has been reported that the relationship between Δ*R*2* and CA concentration is non-linear for pure blood and it has also been predicted that the transverse relaxivity of the CA in blood differs from the relaxivity of brain tissue compartments such as grey and white matter [2–4]. To obtain quantitative values of CBV, CBF, and MTT in absolute terms, an arterial input function (AIF) representing the true concentration of CA in pure blood is needed. Several of the methodological complications associated with quantitative DSC-MRI derive from the difficulties in estimating a reliable AIF owing to, for example, the non-linear relationship between CA concentration and Δ*R*2* and different relaxivities in blood and brain tissue, as mentioned above. However, these relaxivity-related issues have, in most cases, been investigated under the assumption that AIF measurements are made in pure blood, which, in practice, is not normally the case owing to, for example, limited spatial resolution implying that a voxel assumed to represent blood is likely to contain a partial volume of other brain tissue types [5, 6]. In addition, signal saturation (signal clipping) and signal pixel shifts (at low bandwidths) [7] of blood signal are likely to occur at high CA concentrations, and these effects tend to deteriorate the shape of the arterial concentration time curve. Hence, such voxels will typically be excluded from the selected AIF. Instead, a voxel that shows reasonably high response to the CA (in terms of high Δ*R*2*) in combination with an expected shape and early arrival time, will, in practice, normally be used as the AIF. It is thus realistic to believe that most AIFs that are actually registered and judged to be reasonable, in a realistic experimental setting, are biased towards voxels with substantial partial volume effects (PVEs) and a lower mean CA concentration. Although PVEs can affect the magnitude signal in several ways, large PVEs tend, in general, to imply overestimated absolute values of CBF and CBV [1, 3].

To improve the estimation of CA concentration, quantification based on the change in magnetic susceptibility instead of the change in *R*2* has been proposed [8–14]. An altered magnetic susceptibility will alter the phase of the MRI signal, and phase information can directly be used to obtain information about the CA concentration as susceptibility is proportional to CA concentration, and the quantitative relationship between phase and susceptibility is well known for certain geometries [15]. Furthermore, by measuring the phase and performing a deconvolution with the dipole kernel, information about the magnetic susceptibility can be obtained in the general case [16]. The use of magnetic susceptibility for estimations of CA concentration was first introduced for large vessels only, in terms of phase measurements under the assumption of well-defined cylinder geometries [9–11] and, more recently, using quantitative susceptibility mapping (QSM) for general object shapes [8, 12–14]. The advantage of using susceptibility-based methods instead of a Δ*R*2*-based method is the linear relationship between susceptibility change and CA concentration [4, 9, 17]. The proportionality constant, i.e., the molar susceptibility, is known for common gadolinium CAs [18] and quantification of CA concentration in vivo is, in principle, feasible. However, obtaining pixel-wise information about CA concentration in structures of arbitrary shape and orientation relative to the main magnetic field is not straightforward, and QSM is an evolving approach where different algorithms have been proposed to solve the ill-posed problem of using MRI phase information to obtain information about magnetic susceptibility distributions [16]. While promising QSM reconstruction algorithms are continuously developed, there is still need for further evaluation of the performance in challenging geometries [19]. Also, to be able to compare susceptibility values from different measurements or different time points (and thus to quantify CA concentration), a reliable reference region is needed [12]. The amount of artifacts in QSM images is also, in many cases, still more pronounced compared to T2*-weighted magnitude images. Additionally, to perform QSM, in-depth knowledge of phase-data reliability and features of the specific reconstruction method are needed as QSM is not yet a standardized concept.

Due to the well-known problems of using Δ*R*2*-based AIFs and the current difficulties associated with QSM, it is of interest to compare AIFs from these two methods for CA quantification. In this study, Δ*R*2*-based and QSM-based AIFs and tissue ROI curves were thus systematically compared in terms of shape and effect on the resulting CBF and MTT estimates. The AIFs were selected under realistic experimental conditions and image data were acquired in a standard DSC-MRI experiment in healthy volunteers.

## Material and methods

### Subjects and MR imaging

A standard DSC-MRI experiment was performed in 20 healthy volunteers scanned with the same protocol on two different occasions (test–retest) with 7–20 days between the two examinations, using a 3 T MRI unit (Philips Achieva, Philips Medical System, Best, The Netherlands). After bolus injection (5 ml/s injection rate) of a standard dose (0.1 mmol/kg body weight) of CA [Dotarem, Guerbet, Paris, France], followed by a saline flush, a time series of T2*-weighted images (single-shot 2D GRE-EPI) with time resolution 1.24 s, echo time (TE) = 29 ms, matrix size 128 × 128, field of view 220 × 220 mm^2^ and 20 slices of 5 mm thickness with 1 mm slice gap, covering the whole brain, was acquired during the CA bolus passage. A CA pre-bolus with 20% of a standard dose was injected prior to the DSC-MRI protocol as part of a different study [20]. Both phase and magnitude images were obtained. One dataset was excluded from the study, owing to technical difficulties. Informed consent was obtained from all volunteers and the study was approved by the local ethics committee. Administration of gadolinium CA in healthy volunteers on two separate occasions is associated with ethical restrictions, and the data collection scheme was therefore designed to accommodate several independent scientific investigations. Such a multi-study design to maximize the scientific benefit was a prerequisite for obtaining approval by the local ethics committee. Hence, parts of the acquired image data have previously been analyzed for other purposes with clearly separated hypotheses and post-processing approaches [12, 20–23], and in the present study additional and independent image processing and analysis was performed to address new scientific issues.

### Post-processing procedures

Magnitude MRI signal was used to calculate $\Delta R{2}^{*}$ according to

1 $${\Delta }R2^{*} \left( t \right) = - \frac{1}{{{\text{TE}}}} \cdot {\ln}\left( {\frac{S\left( t \right)}{{S_{0} }}} \right)$$

where *S*(*t*) is the signal at time t in the time series of images and S_0_ is the baseline signal.

QSM maps, from which susceptibility-based CA concentration data were extracted, were reconstructed using the morphology enabled dipole inversion (MEDI) algorithm [13, 24–26], using a software package from Cornell University (https://pre.weill.cornell.edu/mri/pages/qsm.html, version 5a with in-house modifications made to adapt to the specific image data used in this project). Projection onto dipole fields (PDF) [24, 27] was used to remove large-scale phase variations in the phase images. As a standard value, for all volunteers, *λ* = 300 was used as the regularization parameter in MEDI and CSF was used as a reference region for the QSM data. More detailed information about the QSM data post-processing has previously been reported [12]. QSM values, assumed to be proportional to the concentration of CA, were obtained as

2 $${\text{QSM}}^{{{\text{conc}}}} = {\text{QSM}} - {\text{QSM}}_{0}$$

where QSM refers to the images in which data were shifted so that the CSF reference was set to zero, and QSM_0_ is the mean of the baseline QSM images.

**Fig. 1:**
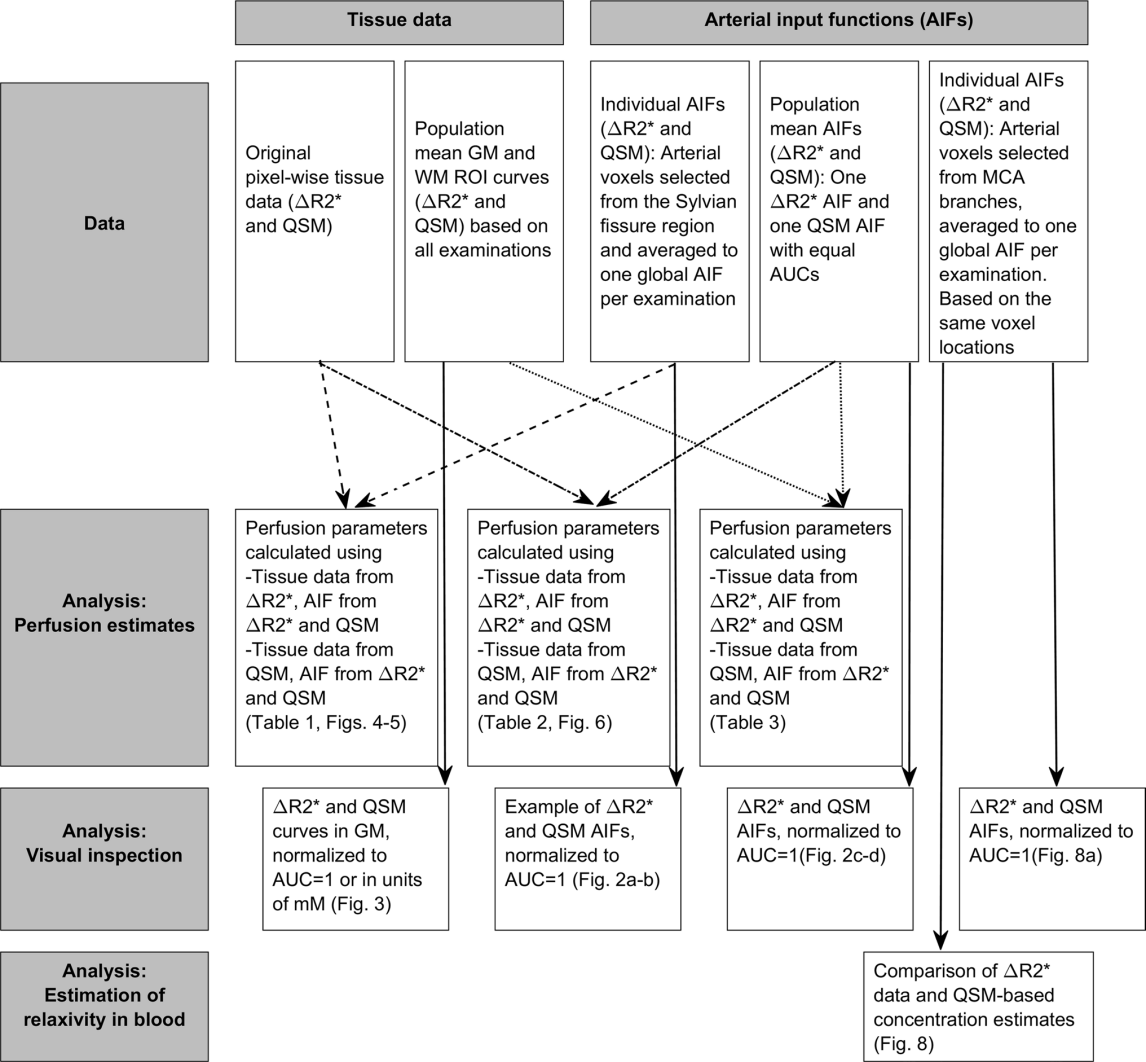
Flowchart showing the combinations of data used to obtain perfusion estimates, tissue curves and arterial input functions for visual inspection as well as AIF voxel relaxivity

All the following steps in the post-processing and analysis procedure are described in detail below and summarized in Fig. 1. The initial selection of potential AIF pixel candidates was made automatically, based on the shape (i.e. no peak distortion), width, and amplitude of the curve as well as on the arrival time of the CA, using a locally developed perfusion software [28]. From the automatically suggested AIF candidates, 4–5 pixels in the Sylvian fissure region (related to CA passage in MCA branches) were chosen to represent the AIF. The same criteria were used to select pixels representing the AIF for both Δ*R*2*-based data and QSM-based data. The included AIF pixels were, however, chosen independently for Δ*R*2*- and QSM-data. For each examination, the mean value of all the chosen AIF pixel time curves was calculated and used as the global AIF for that particular examination. To facilitate a general comparison of the shapes of the AIFs based on Δ*R*2* with those based on QSM, the averaged curves (i.e., the global AIFs) from each individual examination were time shifted such that the initial rise in CA concentration occurred at the same time point for all different examinations in all subjects. These time shifts were determined independently for Δ*R*2*- and QSM-based curves. To assess any overall or systematic difference, the mean value of all examinations was subsequently calculated (referred to as the population mean below), creating one resulting AIF curve based on Δ*R*2* and one curve based on QSM. For the purpose of visual comparison, the curves were normalized such that the area under the curve (AUC) was set to one for both the resulting curves.

For completeness, and to allow assessment of the effect of the AIF on the resulting perfusion estimates, tissue curves from regions of interests (ROIs) were also generated and compared. Grey matter (GM) and white matter (WM) ROIs were generated using SPM8 (https://www.fil.ion.ucl.ac.uk/spm/software/spm8/), based on the magnitude image from the first time point in the DSC-MRI series, including voxels with a respective volume fraction above 0.55. The ROI mean time course was extracted from T2*-weighted as well as QSM images, and Δ*R*2* and QSM^conc^ were subsequently calculated according to Eqs. 1 and 2, respectively. As a result, one tissue curve per tissue type from each examination was obtained and, similarly to the AIFs, the tissue curves were time shifted to match each other, and a mean value of all examinations from all subjects was calculated. This resulted in one population mean Δ*R*2* tissue curve per tissue type and one population mean QSM-based tissue curve per tissue type (i.e., Δ*R*2* as well as QSM curves were based on the mean value of the whole population). GM tissue Δ*R*2* and QSM^conc^ was converted to concentration of CA using

3 $${\text{C}}\left( {\text{t}} \right)_{{{\text{DSC}}}} = \frac{{{\Delta }R2^{*} \left( t \right)}}{{r2^{*} }}$$

and

4 $${\text{C}}\left( t \right)_{{{\text{QSM}}}} = \frac{{{\text{QSM}}^{{{\text{conc}}}} }}{{{\upchi }_{{{\text{mol}}}} }}$$

where *χ*_mol_ is the molar susceptibility. For tissue concentration calculations, the *r*2* used was 85 s^−1^ mM^−1^ [12] and *χ*_mol_ was 308 ppm/M [18]. The population-based mean GM tissue curves originating from ΔR2* data and QSM data were visually compared, both after normalization of the AUC and after conversion to CA concentration. The population mean AIF from QSM was also converted to absolute concentration using Eq. (4) and *χ*_mol_  = 308 ppm/M [18].

To evaluate how the observed overall differences in shape of the two AIF types would affect the result of a pixel-by-pixel DSC-MRI analysis, CBF and CBV were calculated according to Eqs. (5) and (6), respectively:

5 $${\text{CBF}} = \frac{{\left( {1 - H_{{{\text{large}}}} } \right)R_{{\max}} \mathop \int \nolimits_{0}^{\infty } C\left( t \right){\text{d}}t}}{{\rho \left( {1 - H_{{{\text{small}}}} } \right)\mathop \int \nolimits_{0}^{\infty } R\left( t \right){\text{d}}t\mathop \int \nolimits_{0}^{\infty } {\text{AIF}}\left( t \right){\text{d}}t}}$$

6 $${\text{CBV}} = \frac{{\left( {1 - H_{{{\text{large}}}} } \right)\mathop \int \nolimits_{0}^{\infty } C\left( t \right){\text{d}}t}}{{\rho \left( {1 - H_{{{\text{small}}}} } \right)\mathop \int \nolimits_{0}^{\infty } {\text{AIF}}\left( t \right){\text{d}}t}}$$

Here, *ρ* = 1.04 g/ml is the brain density, *H*_large_ = 0.45 and *H*_small_ = 0.25 are hematocrit levels in large vessels and capillaries [29], respectively, *R*(*t*) is the tissue impulse response function and *R*_max_ is the maximal value of this function. Furthermore, MTT was calculated as the ratio of the area to the maximum height of *R*(*t*) [30]:

$${\text{MTT}} = \frac{{\mathop \int \nolimits_{0}^{\infty } R\left( t \right){\text{d}}t}}{{R_{{\max}} }}$$

Calculations with different combinations of tissue data and AIF type (i.e., Δ*R*2* vs. QSM) were performed. For each individual Δ*R*2* tissue dataset, two different individual AIFs, i.e., one Δ*R*2*-based and one QSM-based global AIF corresponding to that particular examination, were used. To allow for an unbiased comparison in the lack of absolute AIF concentration levels, the QSM-based AIF was rescaled to show the same numerical AUC as the Δ*R*2*-based AIF. Similarly, QSM-based tissue data were used with individual AIFs from both Δ*R*2* and QSM. Here, the Δ*R*2*-based AIF was rescaled to show the same numerical AUC as the QSM-based AIF. The AIF rescaling reflects the fact that the amount of CA entering the tissue compartment was the same for Δ*R*2* data as for QSM data, and it implies that the CBV estimates for a given tissue dataset will be independent of the AIF type. Additionally, to limit the potential impact of noise in the AIF on the final results, the whole-population mean AIF curves mentioned above were used, i.e., the mean value of AIFs from all examinations, resulting in only one Δ*R*2*AIF curve and one QSM AIF curve, common for all examinations. Calculations with the same combination of tissue and AIF curves as for the individual AIFs were performed.

All CBF, CBV, and MTT estimates were evaluated in GM and WM ROIs based on segmentation using *new segment* in SPM8 (https://www.fil.ion.ucl.ac.uk/spm/software/spm8/). For some voxels, the concentration time curves based on the QSM images showed a concentration decrease during the CA passage due to artifacts, most frequently seen in WM (possibly caused by the anisotropic susceptibility in WM [31]). This corresponds to negative peak concentrations, prohibiting calculation of physiologically reasonable perfusion parameters. These voxels were thus excluded from the GM and WM ROIs for both Δ*R*2*- and QSM-based data, prior to calculation of mean CBV, CBF and MTT in the ROIs. A two-tailed paired *t* test was performed for CBF and MTT estimates obtained using individual AIFs, comparing results using the same tissue data but AIFs from Δ*R*2*and QSM.

To enable a simplified assessment of potential effects on perfusion estimates caused by differences between Δ*R*2*- and QSM-based tissue data, CBF, CBV and MTT were also calculated using the mean population curves from blood as well as tissue. The same combination of tissue and AIF data was used as for individual tissue data, as described above, but in this case the tissue curves were also rescaled to show the same AUC (implying equal CBV estimates for both Δ*R*2* and QSM). Hence, the QSM-based tissue curve was normalized to show the same AUC as the Δ*R*2* based tissue curve and the QSM-based AIF was normalized to the same AUC as the Δ*R*2*-based AIF.

In QSM reconstruction using MEDI, the estimated susceptibility values and the appearance of the QSM images depends on the regularization parameter *λ*. Hence, the estimated concentration values based on QSM data are also likely to be influenced by the choice of *λ*. To assess the potential importance of this effect, QSM images with different *λ* values (100, 300, 1000, 3000, 5000) were reconstructed for one volunteer and the corresponding AIFs, obtained from the same voxels for each of the QSM image data sets, were visually compared.

In the context of investigating the potential of Δ*R*2* versus QSM data for AIF registration, it is also of some interest to compare the corresponding concentration estimates in the same voxel. Hence, an additional set of AIF pixels were identified in the magnitude images (using the same criteria as described above), and Δ*R*2* data and QSM data from the same voxels were subsequently compared. These data also enabled estimation of the relaxivity for voxels that could potentially be used as AIF voxels in a typical DSC-MRI experiment. In this case, the initial selection (based on Δ*R*2* data) aimed at obtaining as many reasonable AIF candidates as possible, giving a total of 1341 AIFs from all measurements (mean 34, range 5–53, for individual examinations). A visual inspection of both the Δ*R*2* curve and the QSM curve that corresponded to each selected pixel was subsequently performed, and only curves showing an acceptable shape (with a distinct peak and positive values in the CA steady-state period) in both Δ*R*2*-based and QSM-based images were included, leading to a total of 682 AIFs remaining after final selection (mean 17, range 3–26, for individual examinations). By definition, the susceptibility effects of the CA in blood should be confined to the blood compartment in QSM images, and by also including an assessment of AIF shape for the QSM curves, the selected AIF voxels can be assumed to include a blood fraction. To estimate the transverse relaxivity, $r_{2,\text{blood}}^{*},$ for these voxels, the concentration of CA was calculated according to Eq. (4) (using 308 ppm/M [18]). A linear regression of Δ*R*2* versus CA concentration data was performed, with the additional demand that the intercept should be zero, and the relaxivity is thus given by the resulting proportionality constant. One global curve, assumed to represent blood, from each examination was used (representing the mean value of all included curves for that examination), and each time point in the time series (excluding baseline data) corresponded to one data point in the Δ*R*2*-versus-concentration plot.

## Results

**Fig. 2:**
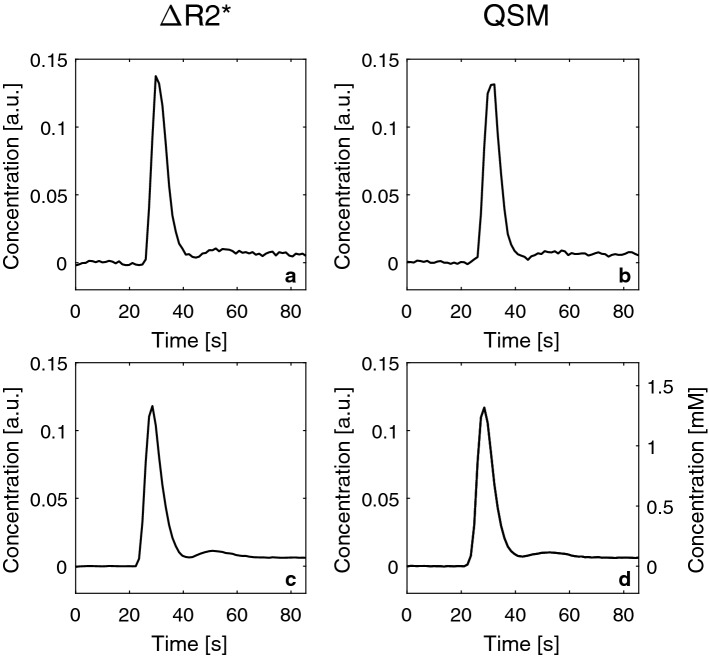
Arterial input functions based on **a** Δ*R*2* data from one examination, **b** QSM data from the same examination as in **a**, **c** mean values of all examinations based on Δ*R*2* and **d** mean values of all examinations based on QSM shown in arbitrary units (left *y*-axis) as well as in units of mM (right *y*-axis)

In Fig. 2, results from the AIF analysis are shown, including representative examples of individual arterial time curves from one examination as well as the whole-population mean Δ*R*2*-based and QSM-based curves. Comparing the whole-population mean curves, when normalized to the same AUC, the curves show very similar appearance. For completeness, the QSM-based whole-population curve is also displayed in terms of absolute concentration.

**Fig. 3:**
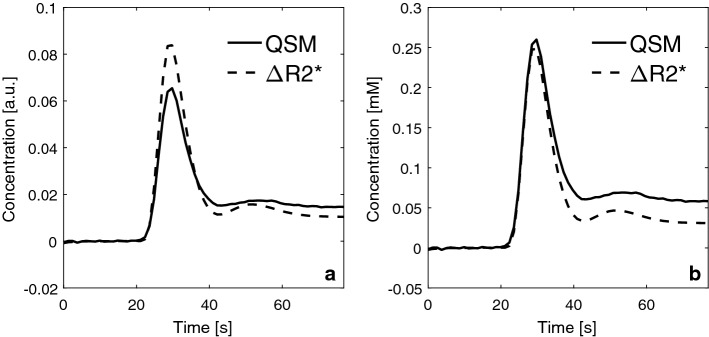
Grey matter tissue curves based on mean values from all examinations, **a** normalized to show the same AUC, and **b** expressed in units of CA concentration [mM] based on the molar susceptibility (for QSM-based curves) and the tissue relaxivity (for Δ*R*2*-based curves)

Results from the tissue concentration analysis are shown in Fig. 3, where the population mean tissue curves, from all examinations, are displayed, rescaled to show the same AUC as well as translated to quantitative concentration values. Compared to the AIF curves, the tissue curves tended to show larger differences between the two quantification approaches.

**Fig. 4:**
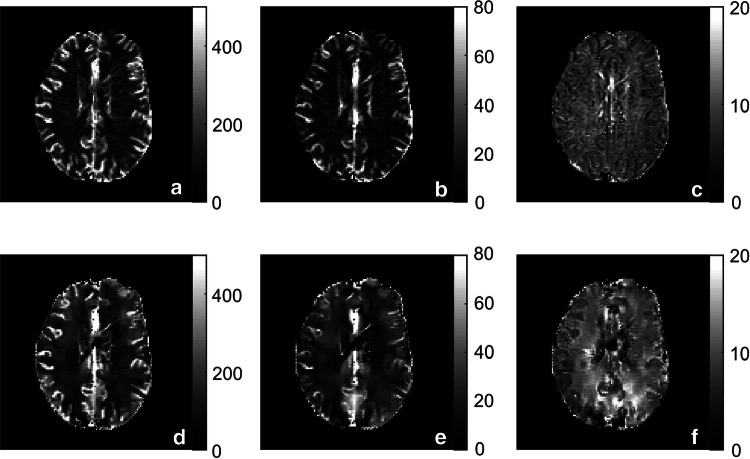
Examples of **a**, **d** CBF, **b**, **e** CBV and **c**, **f** MTT images from one representative volunteer based on individual AIFs. Top row shows maps based on tissue and AIF from Δ*R*2* data and bottom row shows maps based exclusively on QSM data. CBF is expressed in units of ml/(min100 g), CBV in ml/100 g and MTT in seconds. The overestimated values of CBV and CBF, compared to literature, are assumed to be caused primarily by AIF partial volume effects

Figure 4 displays CBF, CBV, and MTT maps calculated with tissue as well as AIF data originating from the same type of quantification approach, i.e., based either entirely on Δ*R*2* data or entirely on QSM data. Overall, the resulting images are similar, but the QSM images seem to suffer from artifacts to a greater extent, and this is particularly obvious in the MTT images.

**Fig. 5:**
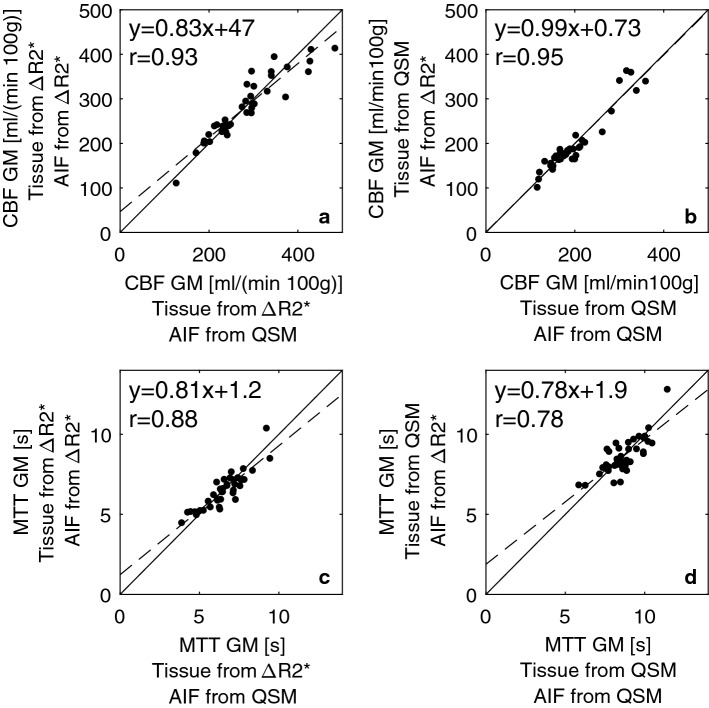
Each column shows a comparison between perfusion estimates obtained using the same tissue data but different AIFs. Left column shows results from Δ*R*2* tissue data and right column results from QSM tissue data. The comparison includes **a**–**b** CBF and **c**–**d** MTT. Results using AIFs from individual examinations are shown. Solid lines indicate the identity line, while dashed lines correspond to the linear regression analysis

**Fig. 6:**
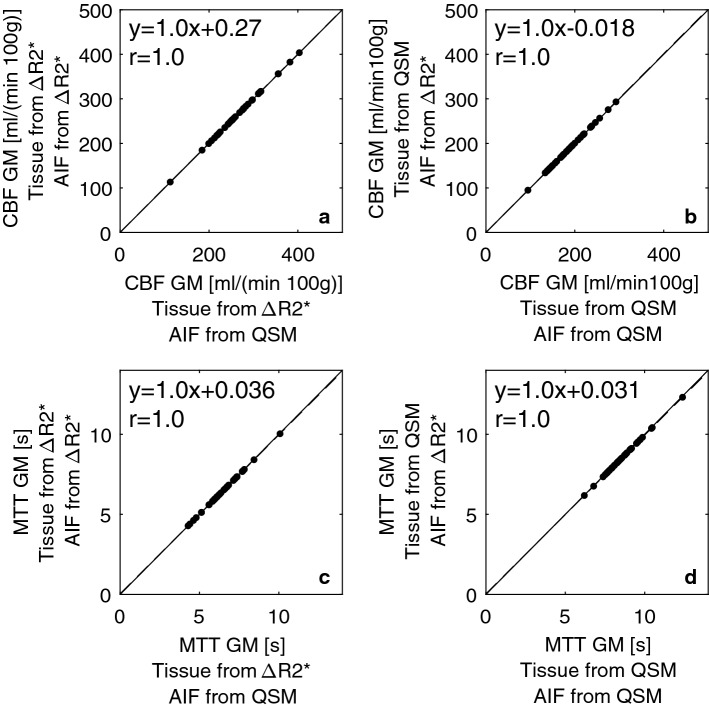
Comparison of parameters resulting from using different AIFs but the same tissue data. In the left column, tissue data from Δ*R*2* are shown while the column to the right shows tissue data from QSM images. Results from the **a**–**b** CBF **c**–**d** MTT analysis using the same AIFs (based on mean values from all examinations) on every single examination are shown. Solid lines indicate the identity line and dashed lines represent the result from the linear regression analysis

In Figs. 5 and 6, results are compared to show differences in the perfusion parameters when using different AIF types. Here, tissue values are based on one concentration quantification method (either Δ*R*2* or QSM) while the AIF is varied to show how the differences in AIF shape can influence the result. In Fig. 5, AIFs from the individual examinations are shown and in Fig. 6, the whole-population mean AIFs, based on Δ*R*2* and QSM, were used for all individual tissue datasets to minimize the risk that CNR differences between the AIF types would influence the results. Note that the two types of AIFs were rescaled to show the same AUC when applied to tissue data from a given quantification approach and, hence, both AIF types are expected to result in the same CBV.

The mean values of all different combinations of tissue data and AIFs are shown in Table 1 (individual AIFs) and Table 2 (whole-population mean AIFs). In general, as expected from the visual appearance of the AIFs, the CBF and MTT values were very similar, regardless of whether AIFs from QSM or Δ*R*2* were used. Furthermore, no significant difference (*p* < 0.05) was observed between the methods, estimated based on individual AIFs. This is consistent with Fig. 5, from which it is also clear that no systematic trend can be observed.

**Table 1:** CBF, CBV and MTT estimates in grey and white matter from calculations based on different combinations of tissue and AIF data

	CBF [ml/(min 100 g)]		CBV [ml/100 g]		MTT [s]	
	GM	WM	GM	WM	GM	WM
Tissue Δ*R*2*, AIF Δ*R*2*	279 ± 71	137 ± 37	29 ± 5	15 ± 3	6.5 ± 1.1	6.6 ± 1.2
Tissue Δ*R*2*, AIF QSM	281 ± 78	138 ± 40	29 ± 5	15 ± 3	6.5 ± 1.2	6.7 ± 1.3
Tissue QSM, AIF QSM	197 ± 62	117 ± 40	26 ± 9	17 ± 7	8.7 ± 1.1	9.5 ± 1.4
Tissue QSM, AIF Δ*R*2*	197 ± 65	117 ± 41	26 ± 9	17 ± 7	8.6 ± 1.1	9.5 ± 1.4

Nominal mean ± standard deviation (SD) of all 39 examinations are shown where AIFs from each individual examination were used

**Table 2:** CBF, CBV and MTT estimates in grey and white matter from calculations based on different combinations of tissue and AIF data

	CBF [ml/(min 100 g)]		CBV [ml/100 g]		MTT [s]	
	GM	WM	GM	WM	GM	WM
Tissue Δ*R*2*, AIF Δ*R*2*	268 ± 56	132 ± 32	28 ± 7	14 ± 4	6.4 ± 1.1	6.6 ± 1.2
Tissue Δ*R*2*, AIF QSM	268 ± 56	132 ± 32	28 ± 7	14 ± 4	6.4 ± 1.2	6.6 ± 1.3
Tissue QSM, AIF QSM	190 ± 43	113 ± 27	25 ± 7	16 ± 5	8.6 ± 1.1	9.4 ± 1.4
Tissue QSM, AIF Δ*R*2*	191 ± 44	113 ± 27	25 ± 7	16 ± 5	8.5 ± 1.1	9.4 ± 1.3

The AIFs used were based on the whole-population mean value of all examinations. Nominal mean values ± SD of all 39 examinations are shown

In Table 3, results from using the whole-population mean tissue curves are shown, and tissue curves were normalized to show the same AUC in the same way as the AIFs. From these results, it is possible to compare the effects of using different tissue data but the same AIFs, unlike the results in Tables 1 and 2.

**Table 3:** Nominal CBF, CBV and MTT estimates from different combinations of tissue curves and AIFs

	CBF [ml/(min 100 g)]		CBV [ml/100 g]		MTT [s]	
	GM	WM	GM	WM	GM	WM
Tissue Δ*R*2*, AIF Δ*R*2*	219	111	26	13	7.2	7.1
Tissue Δ*R*2*, AIF QSM	218	111	26	13	7.2	7.1
Tissue QSM, AIF QSM	166	75	26	13	9.3	11
Tissue QSM, AIF Δ*R*2*	167	75	26	13	9.3	11

Tissue curves as well as AIFs were based on curves generated as mean values from all examinations. The QSM-based curves were normalized to show the same AUC as the Δ*R*2*-based curves

Note that the nominally quantitative CBV and CBF values in Figs. 4, 5, 6 and Tables 1, 2, 3 are generally overestimated compared with literature data obtained with gold-standard techniques. For example, positron emission tomography (PET) measurements by Leenders et al. in healthy subjects resulted in CBF estimates of 52 ml/(min 100 g) in GM and 21 ml/(min 100 g) in WM, and CBV estimates of 5.0 ml/100 g and 2.6 ml/100 g in GM and WM, respectively [32].

**Fig. 7:**
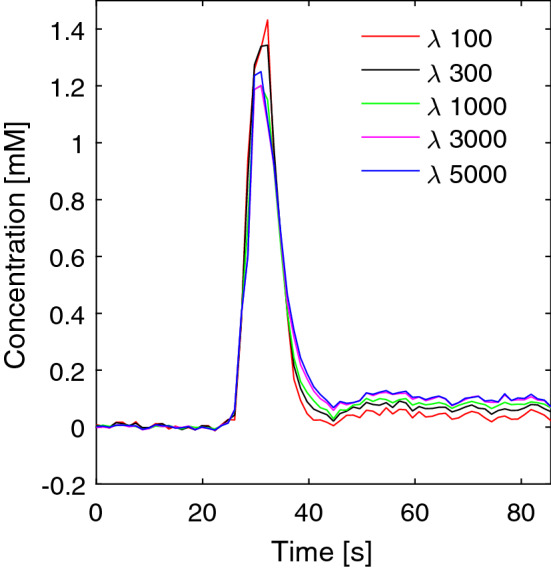
Example of AIFs from one volunteer obtained using different values of the regularization parameter λ in the MEDI QSM reconstruction algorithm. The curves show global AIFs (mean value of 4 voxels) from the same voxels but using different λ values

The resulting AIFs from one volunteer, using different regularization values in the QSM algorithm, are shown in Fig. 7. As expected, the shape is somewhat affected by the choice of *λ*. Note that *λ* = 300 was used for all other results shown in this study.

**Fig. 8:**
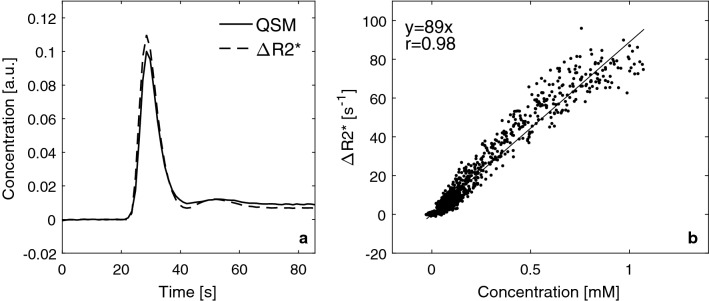
**a** Whole-population mean AIF data (assumed to contain a blood component) corresponding to the same pixel in both Δ*R*2* and QSM images. **b** Δ*R*2* as a function of QSM-based concentration for all the AIF voxels assumed to represent blood. The concentration of CA was estimated based on CA-induced changes in magnetic susceptibility in combination with the molar susceptibility of the gadolinium CA. The AIF voxel relaxivity is estimated to be 89 s^−1^ mM^−1^ based on a linear regression analysis (assuming zero intercept) of the displayed results. The corresponding linear regression analysis results without any prior assumption about the intercept were *y* = 91*x* − 0.99 (*r* = 0.98). Each data point in the plot above represents one time point on the concentration time curve from one examination. All examinations and all time points on the AIF curves (excluding baseline data) are included. It may be of interest to compare the present results with the non-linear relaxivity estimate by Akbudak et al. [2], shown, for example, in Fig. 5b in Ref. [3]

Finally, results from comparing AIF data (assumed to contain a blood component) corresponding to the same pixel in both Δ*R*2* and QSM images are shown in Fig. 8, i.e., the whole-population curves as well as the Δ*R*2*-versus-concentration plot for $r_{2,\text{blood}}^{*}$ estimation. The linear regression showed good correlation, and resulted in a linear relaxivity estimate of $r_{2,\text{blood}}^{*}$ = 89 mM^−1^ s^−1^.

## Discussion

In this study, we report different approaches of using DSC-MRI data for perfusion estimation. Currently, the most common approach in DSC-MRI is to use AIFs based on Δ*R*2* data. However, since numerous drawbacks are associated with this approach, it was regarded to be of value to investigate the potential use of AIFs based on QSM data.

An initial visual comparison of the AIFs based on Δ*R*2* and QSM data indicates that there is no substantial systematic difference in shape between the two curves, and if the non-linear relationship between Δ*R*2* and CA concentration, presented by Akbudak et al. [2] for whole blood, were to be applied to the experimental Δ*R*2* data, the difference in appearance between the two curves would increase dramatically. Our experimental results thus imply that application of previously published non-linear relationships between Δ*R*2* and concentration in arterial blood [2] are inappropriate for AIFs selected according to common criteria in a realistic DSC-MRI experiment. The main reason for this is, most likely, that voxels selected for AIF purposes in this study do not correspond to a compartment consisting of blood only, and the concentration estimates are thus assumed to be largely affected by static brain tissue. A difference between the results in this study and the results by Akbudak et al. [2] was thus quite expected.

Hence, although the observed AIFs are assumed to reflect effects of CA in arterial blood, the measured response is likely to be augmented by the presence of a static brain tissue component [5]. It should in this context be noted that careful selection of AIF locations, both in magnitude and phase images, has been shown to allow for selection of AIFs with correct shape (but underestimated AUC) also at some distance from the blood compartment [33, 34]. However, as was found in studies by both Kjølby et al. [5] and Bleeker et al. [33], a short echo time is preferred to be able to register a correct shape of the AIF. For longer echo times PVEs will, in general, lead to a broadening of the AIF curve [5], but the correct shape can still be found, albeit at a location further away from the vessel [33].

Figures 2d and 7 indicate that the maximal mean concentration levels in AIF voxels were of the order of 1 mM (compared with approximately 0.25 mM in tissue, cf. Fig. 3b), and previous studies have indicated that true peak concentrations in large vessels are expected to be of the order of 5–10 mM [8, 18]. Hence, AIFs with reasonable shape, measured in a realistic DSC-MRI experiment, seem to originate predominantly from voxels positioned partially or (for Δ*R*2* data) entirely outside the actual blood compartment, as indicated by the low maximal concentration levels in this study. Hence, in a standard DSC-MRI experiment, without, for example, calibration/rescaling using supplementary data or a separate AIF measurement in a large brain-feeding artery, the possibility to accomplish absolute quantification of CBV as well as CBF is, in effect, non-existing because a successful estimation requires that the AIF concentrations represent a voxel that contains 100% blood. To address this problem, Kellner et al. [35] measured arterial concentration in an additional slice, interleaved with the standard DSC-MRI sequence, with parameters optimized to measure blood, to obtain the AIF from the carotid arteries. With this method, AIFs with a reasonable maximal concentration (approximately 8–10 mM) were observed. As expected, the maximum concentration in the study by Kellner et al. [35] was much higher than in the present study because they measured in larger vessels with less PVEs. Kellner et al. [35] stated that the AIF obtained in the carotid arteries could be expected to show less dispersion as it is measured upstream in the arterial vessel tree. When comparing the curves from Kellner et al. [35] with the AIFs in this study, no obvious difference in AIF width could be seen (cf. Fig. 2d), although it is, obviously, difficult to make any detailed comparison between different groups of subjects.

In the presence of PVEs in selected AIF voxels, it is clear that a potential additional problem is the shape of the AIF and the question whether Δ*R*2*-based AIFs are more prone to PVE-induced distortions in shape than susceptibility-based AIFs. Our results show that, when comparing the curves visually, the systematic difference between Δ*R*2*-based and QSM-based AIFs is small and the shapes of the curves are indeed very similar. Obviously, both the Δ*R*2*- and the QSM-based AIFs suffer from PVEs, and both types of data can thus be incorrect. It is, however, unlikely that PVEs will distort the shape of the AIF in the same way for both Δ*R*2*- and QSM-based data, which makes the current results relevant.

Considering that the effect of the CA in the QSM images is assumed to be directly proportional to the concentration, susceptibility-based AIF registration may have advantages. However, it is not straightforward to objectively compare the shapes of two different curves, and it is not entirely easy to understand the consequence of the difference in shape when the respective AIFs are used in the deconvolution procedure. In this study, the areas under the AIF curves were set to be equal, leading to the same CBV being estimated independently of the AIF used, thereby highlighting differences in the shape-dependent parameters CBF and MTT. When the same tissue data were used with different AIFs, very similar results were obtained. Hence, it seems that, in practical measurements, CBF and MTT estimates based on Δ*R*2* and QSM AIFs are very similar, which is of interest to note considering the substantial concerns that have been expressed with regard to Δ*R*2*-based AIFs.

As shown in previous studies, both magnitude-based and phase-based AIFs are influenced by PVEs [4–6]. As PVEs alter the complex MR signal, the phase data used to reconstruct QSM images are indeed affected and PVE effects will thus influence the QSM results as well. For magnitude-based AIFs, the shape can differ substantially depending on the characteristics of the signal from the surrounding tissue. Although a low fraction of blood in the voxel will generally lead to underestimated ΔR2* at peak concentration [5], PVEs can, depending on circumstances, lead to either underestimated or overestimated peak Δ*R*2* values as well as high Δ*R*2* values at time points that do not coincide with maximal concentration. These spuriously high values correspond to situations when the vector sum of the signal vectors from surrounding tissue and blood add up to a signal close to zero and thus an apparently very high Δ*R*2* value [5, 6]. The estimated CBF values also depend on the expected maximal signal drop, where a larger maximal signal drop corresponds to a higher sensitivity to PVEs [36]. Additionally, even a small fraction of tissue in the voxel chosen to represent the arterial signal can have a substantial effect on the resulting CBF value due to the complex addition of signals from two compartments affecting the shape of the AIF [36]. For phase-based AIFs, on the other hand, the shape of the curve has been shown to be less sensitive to PVEs [6, 34, 37]. For a cylinder parallel to the main magnetic field, the phase effect is proportional to the CA concentration, with a theoretically known proportionality constant, at least for concentrations to be found in vivo [6]. The phase is generally proportional to the concentration of CA (but with different proportionality constants depending on geometry), and even for vessels perpendicular to the main magnetic field, it has been shown that phase information provides a larger number of voxels with a reliable shape compared to magnitude-based AIFs [34]. However, the phase PVEs for a cylinder are complicated and depend on the relationship between the radius of the cylinder, the in-plane voxel dimension as well as the slice thickness [38]. For example, in situations where the slice thickness is four times larger than the in-plane voxel resolution, and the size of the vessel is about the same as the in-plane resolution, a phase shift with opposite sign to what is expected can be seen in a voxel containing a vessel that is perpendicular to the main magnetic field [38]. In the present study, the reconstructed magnetic susceptibility was used instead of the directly measured phase, but phase errors caused by PVEs will, in one way or another, affect the susceptibility map as well, although the exact manifestation is difficult to predict without further investigation. Haacke et al. [39] showed that the magnetic susceptibility calculated by QSM was indeed affected by PVEs and the magnetic susceptibility was shown to be underestimated in a cylinder oriented perpendicular to the main magnetic field even for cylinders with quite large diameters in a voxel with an aspect ratio of 1:4.

Tissue curves tended to show larger differences in shape between Δ*R*2* and QSM data than did the AIFs, and depending on whether the curves were rescaled to show equal AUCs or converted to concentration levels, the curves differed the most at different periods during the time series. When converted to concentration of CA, the major difference between the curves was seen during the CA steady-state period, after the first bolus passage. It is possible that the effect of the CA on Δ*R*2* differs between different concentrations in tissue, because the diffusion length relative to the CA-induced magnetic field inhomogeneity will be concentration dependent [40, 41]. Hence, different models have been used to describe the relaxation effects during the passage of CA in tissue, i.e., large vessels and capillaries with high CA concentration levels are described by the static dephasing regime (SDR) and capillaries with low CA concentration are best described by the diffusional narrowing regime (DNR) [40, 41]. As a consequence, a weaker dephasing effect can be expected in tissue at low CA concentrations which could explain the lower estimated concentration levels (compared to QSM-based values) after the main bolus passage, as seen in this study (Fig. 3b) [40, 41]. However, simulations performed by Kjølby et al. [3] predicted a linear relationship between CA concentration and Δ*R*2* in tissue for GRE sequences, over a relevant range of CA concentrations, and this linearity was thus assumed to be valid in this study. It should also be noted that, in this study, no consideration was taken to the recirculation of CA because simulations by Kosior et al. [42] have predicted that there is no need to remove the effect of recirculation if the CA response is linear to the concentration. Although the tissue relaxivity issue has been thoroughly investigated [40, 41, 43–45], QSM data are still relatively unexplored in the context of dynamic contrast-enhanced perfusion measurements. Hence, future studies comparing Δ*R*2* data to QSM data in tissue will most likely be of interest. When using the tissue curves, rescaled to show the same AUC, for CBF and MTT calculations, the results showed 31% higher CBF values and up to 23% lower MTT values in GM when using tissue curves from Δ*R*2* compared to QSM-based tissue curves. Hence, in this study, the resulting CBF and MTT estimates were clearly influenced by the differences in tissue-curve shape associated with the choice of tissue CA concentration quantification approach. In this part of the study, only the curves based on the mean value of the whole population were used in the analyses. A similar methodology would be feasible also for the individual examinations, by normalizing the tissue curves pixel-by-pixel. However, such an approach would also affect the entire QSM-based CBF, CBV, and MTT maps (i.e., if all curves were to be normalized to Δ*R*2* data) and for that reason, only the AIFs were mutually normalized in the part of the study where the effects of different AIFs were analyzed.

When inspecting the perfusion maps based exclusively on Δ*R*2* and QSM, respectively, the overall appearance of the images is quite similar between the two quantification methods. This implies that the same physiological parameters are indeed measured; an observation which is further strengthened by the other results of this study, and this implies that the compliance between the different approaches is generally good. However, especially in the displayed MTT map, it is clear that parameter maps based on data from QSM tend to contain more artifacts. However, since the pulse sequence used in this study was a conventional DSC-MRI sequence optimized for Δ*R*2*-based perfusion images, the QSM-based images can most likely be improved using a different pulse sequence, optimized for QSM measurements (i.e., multi-TE 3D GRE with high spatial resolution). Obviously, future development and optimization of available QSM reconstruction methods is also likely to improve QSM image quality in general terms.

Some difficulties are encountered when using QSM for susceptibility quantification. One major issue is the need for a reference tissue to be able to compare QSM values from different points in time. The initially obtained QSM images, from the MEDI toolbox, are not displayed in units of absolute susceptibility, due to, for example, B0 drift of the scanner during the measurement and the fact that the absolute phase information is lost during the QSM reconstruction procedure [46]. Hence, to compare QSM values from different points in time, a reference is needed that is unaffected by the CA during the dynamic QSM imaging. It is indeed a challenge to find a stable reference compartment without any large artifacts, not affected by the CA, and the choice of voxels used as a reference will influence the resulting susceptibility estimates and hence the quantification of CA concentration. Another intrinsic issue, when using the MEDI algorithm for QSM reconstruction, is that the setting of the regularization parameter will affect the susceptibility values and thus also the estimated concentrations. Hence, the shape of the AIF will also be affected by the choice of *λ* as illustrated by an example in this study. The appropriate choice of *λ* depends on the data, and in this study *λ* = 300 was chosen as a tradeoff between reducing artifacts in the images and obtaining the correct susceptibility values. Using a different *λ* would obviously alter the results of this study, although the observed differences in AIF shape between different *λ* values were not huge.

To enable a parameter comparison at comparable AIF noise levels, CBF and MTT values from population mean AIF curves with very low noise levels (for both Δ*R*2* and QSM) were calculated. For the original, noisy AIFs, similar values were obtained and, interestingly, the same overall pattern was seen independently of the noise level in the AIFs (cf. Figs. 5 and 6).

To estimate the relaxivity in voxels that could be used as AIFs (assumed to represent blood), Δ*R*2*-based and QSM-based curves from the same location (i.e., the same pixels) were compared. The relaxivity was found to be 89 mM^−1^ s^−1^, based on a linear regression analysis of Δ*R*2* versus QSM-based CA concentration, which is significantly different from the relaxivity (i.e., Δ*R*2*-vs-concentration) relationship found in whole blood by Akbudak et al. [2]. The data in the present study were heavily influenced by PVEs and a deviation in relaxivity from that of whole blood was thus quite expected. However, it is interesting to note that the estimated relaxivity was instead very similar to the relaxivity previously observed in tissue by Lind et al. [12], and it is also consistent with the tissue relaxivity predicted in simulations by Kjølby et al. [3]. The presence of arterial PVEs would, intuitively, result in a relaxivity that is more similar to tissue relaxivity, but, considering that the observed mean concentrations of CA were much higher in the AIF voxels than in tissue, PVEs do not seem to fully explain the apparent close similarity in relaxivity between the AIF voxels representing blood and tissue voxels. When considering the whole-blood data by Akbudak et al. [2] in the context of the present results, it should be remembered that the relaxivity of whole blood is concentration dependent and increases with concentration. As the mean concentration in tissue is much lower (depending on CBV) than in whole blood, the relaxation effect (i.e., Δ*R*2*) in tissue should, at a given point in time during the bolus passage, realistically be compared to the relaxation effect in whole blood at much higher concentrations. For increasing concentrations, the ratio of Δ*R*2*_tissue_ to Δ*R*2*_blood_ decreases, and the relaxation effect of whole blood approaches the relaxation effect in tissue (cf. Fig. 5 in Ref. [3]). However, in this study, the observed mean concentration of voxels representing blood was considerably lower than the concentration expected for whole blood (as confirmed by the QSM data, cf. Fig. 2d) due to PVEs. Hence, CBF and CBV were accordingly overestimated, due to underestimated AIF voxel concentrations (cf., Eqs. 5, 6).

## Conclusion

In this study, AIFs selected by following a common practical procedure, using data from a standard DSC-MRI protocol, were studied, and AIFs based on Δ*R*2* and QSM images were compared. Visually, no distinct differences in the shape of the AIFs were found and the resulting perfusion estimates were very similar. The relaxivity of voxels containing blood (89 mM^−1^ s^−1^) was similar to literature values of tissue relaxivity.
